# Supplementary material for: Exploring the Anti-Colorectal Cancer Mechanism of Norcantharidin Through TRAF5/NF-κB Pathway Regulation and Folate-Targeted Liposomal Delivery
Source: Int J Mol Sci. 2025 Feb 9;26(4):1450. doi: 10.3390/ijms26041450 (PMC11855010; doi:10.3390/ijms26041450)
Supplement: Supplementary file 1 [file ijms-26-01450-s001.zip › ijms-3436684-supplementary.pdf]

Supplementary Materials

Exploring the Anti-colorectal Cancer Mechanism of Norcantharidin Through TRAF5/NF-κB Pathway Regulation and Folate-Targeted Liposomal Delivery

Fanqin Zhang[1†], Xiaodong Chen[1†], Chuanqi Qiao[1], Siyun Yang[1], Yiyan Zhai[1], Jingyuan Zhang[1], Keyan Chai[1], Haojia Wang[1], Jiying Zhou[1], Meiling Guo[1], Peiying Lu[1], Jiarui Wu[1,*]

[1]Department of Clinical Chinese Pharmacy, School of Chinese Materia Medica, Beijing University of Chinese Medicine, Beijing 102488, China

[†] These authors contributed equally to this work.

[*]Address correspondence to:

Jiarui Wu, email: exogamy@163.com ;

Contents

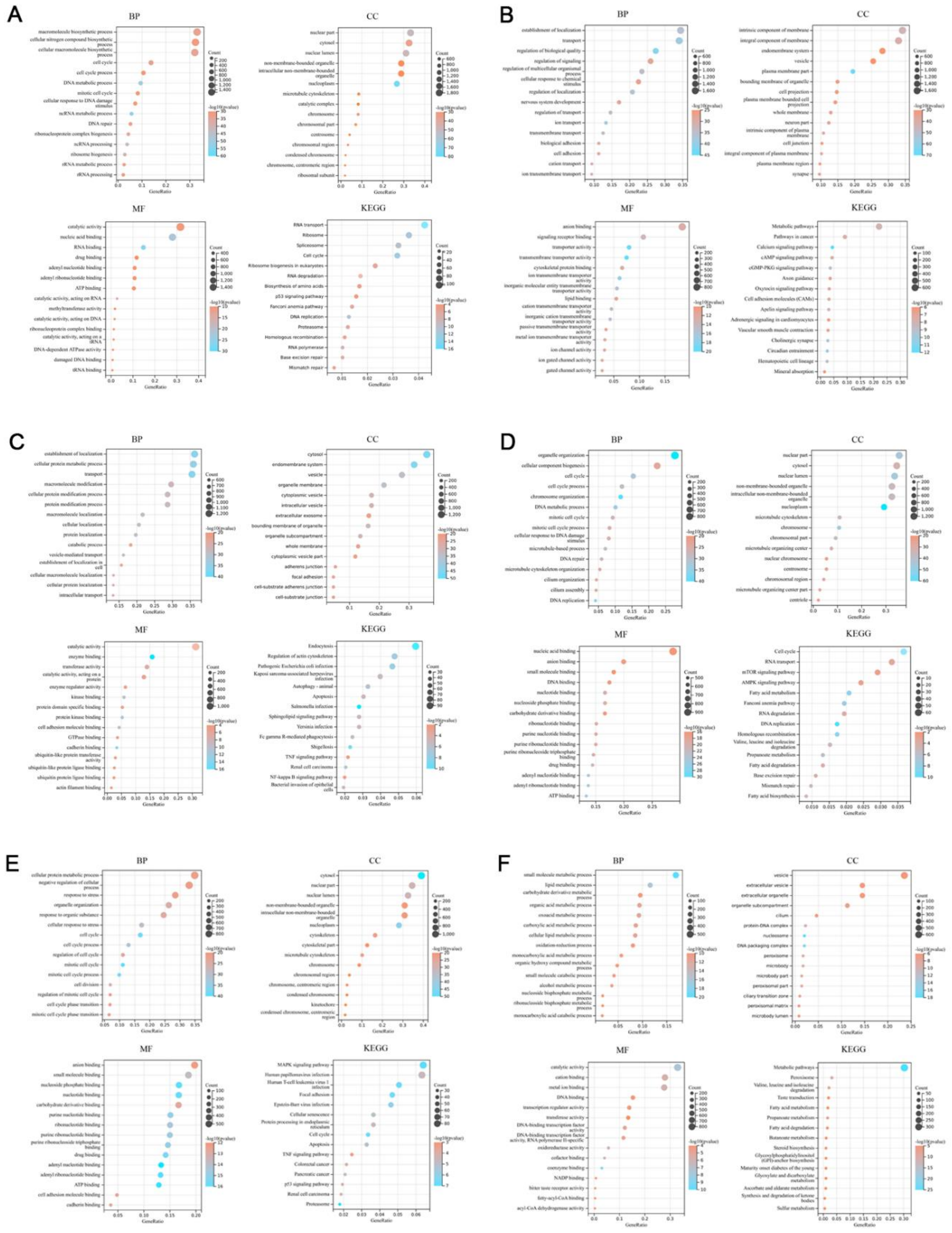

Figure S1. GO and KEGG enrichment analysis were performed for up-regulated and down-regulated genes in tumor samples (A, B), in HCT116 (C, D) and HT-29 (E, F).

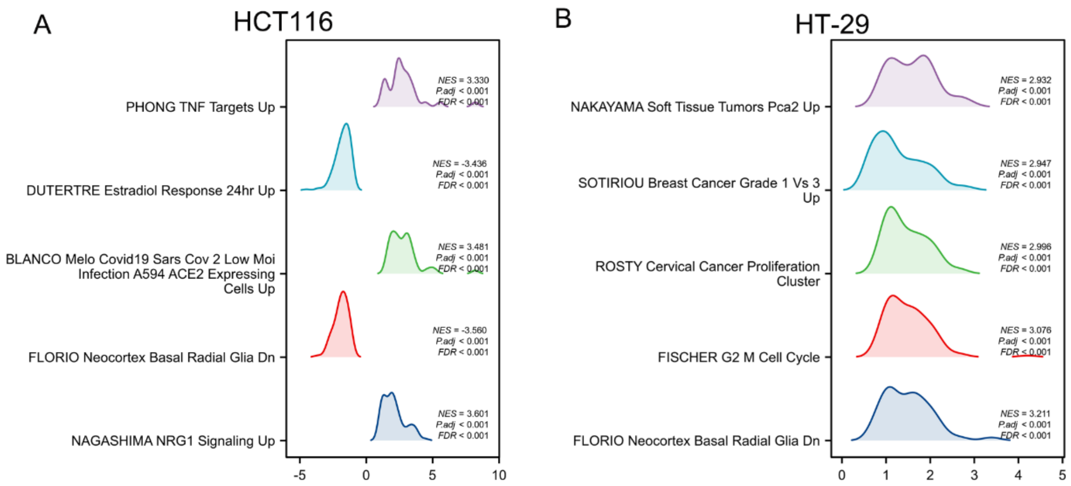

Figure S2. The top five gene sets ranked by Normalized Enrichment Score (NES).

Table S1 The different preparation conditions of the liposome of NCTD encapsulation rate (%)（n=3，$\bar{x} \pm SD$）

A: B: C	10 min	20 min	30 min	60 min
1: 1: 20	21.7 ± 2.7	25.5 ± 2.4	25.1 ± 2.7	24.7 ± 1.8
2: 1: 20	24.4 ± 1.3	30.1 ± 3.0	31.2 ± 3.3	30.7 ± 3.7
3: 1: 20	29.6 ± 1.8	34.1± 3.1	34.2 ± 3.1	35.7 ± 4.1
5: 1: 20	33.1 ± 4.3	39.4 ± 2.9	43.6 ± 2.2	39.6 ± 3.4

A, B, and C represent the mass of cholesterol, norcantharidin and DSPE-PEG_{2000}, respectively.

Table S2 The different preparation conditions of the liposome of FA-NCTD encapsulation rate (%)（n=3，$\bar{x} \pm SD$）

A: B: C: D	10 min	20 min	30 min	60 min
1: 1: 10: 10	24.0 ± 2.8	27.8 ± 3.5	27.7 ± 2.6	28.1 ± 2.6
2: 1: 10: 10	26.5 ± 4.0	32.9 ± 2.5	35.1 ± 1.8	31.6 ± 1.6
3: 1: 10: 10	30.9 ± 3.4	39.8 ± 2.9	37.1 ± 2.1	34.9 ± 2.0
5: 1: 10: 10	36.1 ± 3.1	41.8 ± 1.7	45.8 ± 2.9	37.8 ± 2.3

A, B, C and D represent the mass of cholesterol, norcantharidin, DSPE-PEG_{2000} and DSPE-PEG_{2000}-FA, respectively.
